# First-time detection of bovine viral diarrhoea virus, BVDV-1, in cattle in Botswana

**DOI:** 10.4102/ojvr.v86i1.1764

**Published:** 2019-10-09

**Authors:** Sara Lysholm, Solomon S. Ramabu, Mikael Berg, Jonas J. Wensman

**Affiliations:** 1 Department of Clinical Sciences, Faculty of Veterinary Medicine and Animal Science, Swedish University of Agricultural Sciences, Uppsala, Sweden; 2 Department of Animal Science and Production, Botswana University of Agriculture and Natural Resources, Gaborone, Botswana; 3 Department of Biomedical Sciences and Veterinary Public Health, Faculty of Veterinary Medicine and Animal Science, Swedish University of Agricultural Sciences, Uppsala, Sweden

**Keywords:** bovine viral diarrhoea virus, BVDV-1, cattle, Botswana, infectious diseases

## Abstract

Infectious diseases are serious constraints for improving livestock productivity. Bovine viral diarrhoea virus (BVDV) is a virus causing grave economic losses throughout the cattle producing world. Infection is often not apparent, but the virus can also cause respiratory signs, diarrhoea, reproductive problems and immunosuppression. Risk factors for disease transmission include, but are not limited to, herd size, animal trade and grazing on communal pastures. Several prevalence studies have been conducted in southern Africa, but in Botswana the occurrence is largely unknown. In this study, blood samples were obtained from 100 goats from three villages around the capital city, Gaborone. Also, 364 blood samples from cattle around Gaborone, collected as part of another study, were analysed. The detected antibody prevalence was 0% in goats and 53.6% in cattle when using a competitive enzyme-linked immunoassay. Three animals from two different herds were positive for viral nucleic acids on polymerase chain reaction. The two herds with viraemic animals had significantly higher antibody prevalence compared to the other herds. Also, two of the detected viruses were sequenced and found to be most similar to BVDV-1a. To the authors’ knowledge, this is the first time that sequencing has been performed on BVDV isolated in Botswana.

## Introduction

Livestock play a vital role in alleviating poverty and hunger for numerous farmers around the world, for example, in countries such as Botswana in southern Africa, where 81.5% of the cattle and 95% of the goats are kept by traditional farmers (Statistics Botswana 2016). It is a well-known fact that in order to optimise livestock performance, it is crucial to maintain a good animal health status. A major obstacle in this regard is infectious diseases. Bovine viral diarrhoea virus (BVDV) is a positive-sense single-stranded ribonucleic acid (RNA) virus in the genus *Pestivirus*, which can have a grave exacerbating effect on animal health and productivity (Houe, Pedersen & Meyling 1993). There are two different genotypes, BVDV-1 and -2, and within these several subgenotypes, exists (Fulton et al. 2003; Vilcek et al. 2001). Cattle are considered the primary host, but serological evidence of infection has also been found in, for example, sheep, goats and eland antelope (Broaddus et al. 2007; Torsson et al. 2017; Vilcek et al. 2000). Bovine viral diarrhoea virus infection is in most instances subclinical, but the virus can also cause respiratory signs, diarrhoea, reproductive failure, congenital malformations, pyrexia, depression, inappetence, nasal discharge and erosion of oral mucosa (Walz 2015). The virus is also known to cause leukopenia and immunosuppression which, in turn, renders the animal more susceptible to secondary infections. However, the most serious outcome of infection is the development of persistently infected foetuses (Walz 2015).

Bovine viral diarrhoea virus is present in the majority of countries and seroprevalence is often 60% or more in endemic regions (Houe & Meyling 1991). Differences in seroprevalence are dependent on various factors, for example, on trade routines, usage of communal pastures as well as vaccination practises (Houe 1999). Antibody prevalence is generally higher in adults compared to young animals (Houe & Meyling 1991; Hyera, Liess & Frey 1991; Mishra et al. 2009; Nigussie et al. 2010; Torsson et al. 2017), in large than in small herds (Almedida et al. 2013; Graham et al. 2013; Mockeliuniene et al. 2004; Sarrazin et al. 2013) and is often elevated in areas where cattle density is high (Saa et al. 2012). For small ruminants, regular contact with cattle has been shown to constitute a risk factor (Mishra et al. 2009). Beside this, seropositivity is significantly higher in herds with persistently infected animals compared to herds with only transient infection (Houe & Meyling 1991). In herds with persistent infection, where the animals are kept under close confinement, antibody prevalence is usually 90% or more by the time that the persistently infected calf has reached 3–4 months of age (Houe et al. 1993). The prevalence of persistently infected animals in endemic areas is also highly variable but is generally around 1–2% (Houe & Meyling 1991).

Several prevalence studies have been conducted in southern Africa. In South Africa, prevalences ranging from 37% to 100% in cattle have been detected (Ferreira, Lourens & Van Vuuren 2000; Njiro et al. 2011; Ularamu et al. 2013). In a Namibian study in the late 1980s, 49% of cattle, 9% of sheep and 5% of goats had neutralising antibodies to BVDV (Depner, Hubschle & Liess 1991b). On the Kafue flats in Zambia in 1987, an antibody prevalence of 76.2% in cattle was found (Ghirotti et al. 1991). In Tanzania, a 34% seroprevalence in cattle, 32.1% in sheep and 24.9% in goats have been observed (Hyera et al. 1991). More recently, Torsson et al. (2017) found 3.9% and 1.7% calculated true prevalence in sheep and goats in Tanzania. In Botswana, however, knowledge of BVDV prevalence is limited and dated. In the 1970s, two cattle herds with clinical signs indicative of BVDV infection were investigated, and an antibody prevalence of 42% and 70% found. Also, testing was conducted in a nearby village without clinical signs of BVDV, which detected a 19% seropositivity (Hunter & Carmichael 1975). In another study in 1973, 88 out of 100 Botswana cattle were seropositive for BVDV (Theodoris, Boshoff & Botha 1973, referred by Depner et al. 1991b).

Several different genotypes of BVDV have been detected in Africa and BVDV-1a is the genotype most frequently found (Yesilbag, Alpay & Becher 2017). To the authors’ knowledge, sequencing of BVDV has never been undertaken in Botswana. Hence, the aim of this study was to study the seroprevalence of BVDV in cattle and goats around Gabarone, and to identify circulating genotypes.

## Research method and design

### Study design

Blood samples from 100 goats were collected in three villages situated in or just outside of Gaborone, Botswana, namely Modipane, Kopong and Gakuto. Samples were obtained in September 2016, which is at the end of the dry season. Five smallholder farmers in Modipane, three in Kopong and three in Gakuto were chosen for the study. Inclusion criteria for farmers were residing in the village of choice, owning an appropriate number of adult goats, that is, 20–30 goats, which is equal to the average herd size in Botswana (Statistics Botswana 2016), as well as on consenting to participate in the study. Ten goats from each herd were sampled in all flocks except two in which the total number of adults was less than 20. As these two herds were located immediately adjacent to each other and the animals usually grazed together, they were considered as one epidemiological unit, and sampling hence divided between the two, that is, five goats from each herd. Only adult goats (> 1 year old) were selected and both sexes were represented; however, as all flocks consisted of more female goats than male goats, no effort was made to sample both sexes evenly. Selection of goats was randomised by catching all adults in a flock but only sampling every second or every third animal, depending on the size of the flock.

Jugular blood samples were obtained using a closed vacutainer system and serum tubes (BD Vacutainer^®^, Franklin Lakes, New Jersey, United States [US]). A short description of each animal was recorded, as well as an assessment of their body condition score (BCS). Body condition score was estimated by inspecting the goats visually as well as palpating the lumbar and sternal area, and then assigning them a number from 1 to 5, one being extremely thin, five obese and three ideal. After sampling, the tubes were left standing in room temperature to allow sera to separate. Serum was transferred and then stored at -20 °C until further use.

In addition, 364 blood samples from cattle (> 6 months) previously obtained in October 2014 to March 2015, that is, the Botswana warm season, were tested (Ramabu et al. 2018). Samples were taken within an approximate 150 km radius of Gaborone from both beef and dairy cattle. Nine different farms, five dairy and four beef, were chosen for the study. In dairy farms, samples were obtained from the whole herd, whereas in beef farms, the farmer selected a paddock and samples were then taken there. Approximately 75% – 100% of the animals at each farm or enclosure were sampled. After sampling, the test tubes were left standing in order to allow serum to separate. Serum was then placed in cryotubes and stored at -20 °C until further use.

### Antibody enzyme-linked immunoassay

For detection of antibodies, a competitive enzyme-linked immunoassay (ELISA) was used (sensitivity 98.9%, specificity 100% on bovine samples according to the manufacturer, data for goats not available; IDEXX BVDV/MD/BDV p80 Protein Antibody Test Kit, IDEXX, Hoofddorp, The Netherlands). The test was used according to the instructions provided by the manufacturer. The absorbance values were measured at 450 nm (Thermo Scientific Multiskan FC ELISA reader, Thermo Scientific, Vantaa, Finland). Then, the validity of the positive and negative controls was calculated. For invalid assays, the assay was not repeated because of shortage of material. Percentage inhibition was computed by dividing the sample absorbance value with the mean of the negative control, and then multiplying the result with 100. Samples with a higher value than 50% were considered negative and lower than 40% were considered positive. Samples with a value between 40% and 50% were denoted as doubtful.

### Antigen enzyme-linked immunoassay

Sera negative on antibody ELISA (Ab-ELISA) were subsequently analysed with antigen ELISA (Ag-ELISA) (sensitivity approaching 100%, specificity 99.7% according to the manufacturer; IDEXX Bovine Viral Diarrhea Virus Antigen Test Kit/Serum Plus, IDEXX). The test was performed according to the instructions provided by the manufacturer. The plate absorbance was measured at 450 nm (Thermo Scientific Multiskan FC ELISA reader, Thermo Scientific, Vantaa, Finland). Validity of the negative and positive control was calculated. Percentage inhibition was computed by subtracting the mean of the negative control from the absorbance value of the samples. The result was interpreted as negative if the difference was less than 0.3, but positive if it was more than 0.3.

### Ribonucleic acid isolation

Ribonucleic acid was isolated from samples positive for antigen on Ag-ELISA, as well as from serum samples negative for antibodies and antigen in herds with a seroprevalence of 50% or more. Unfortunately, the remaining sample volume was too low in some samples and hence only 26 samples were subjected to RNA extraction. The procedure was performed according to the instructions provided by the manufacturer (Macherey-Nagel Viral RNA isolation Nucleospin RNA Virus kit, Macherey-Nagel, Düren, Germany). The isolated RNA was stored at -20 °C until further use.

### Find The Agent cards

All samples subjected to RNA isolation were subsequently applied to Whatman Find The agent (FTA) cards (Sigma-Aldrich, Saint-Louis, Missouri, US) to enable transportation. From each sample, 125 *µ*L of serum, 30 *µ*L RNA-isolate and 10 *µ*L polymerase chain reaction (PCR) product were applied.

### Reverse transcriptase polymerase chain reaction

Polymerase chain reaction was subsequently performed on the PCR product that had been applied to FTA cards. The cards were placed on a punch pad and 2 mm punches were obtained using a Harris micro punch (Sigma-Aldrich). One punch from each sample was then subjected to elution. In this step, the punch was placed in a microcentrifuge tube, containing 51 *µ*L RNA-processing buffer (50 *µ*L buffer containing 10 mM Tris-HCl and 0.1 mM ethylenediamine tetraacetic acid, and 1 *µ*L dithiothreitol) and was subsequently left to incubate for 15 minutes, after which the punch was removed. Polymerase chain reaction was then performed on both the eluate as well as on unprocessed punches directly included in the PCR tube during the reaction (Applied Biosystems AgPath-ID One-Step RT-PCR Reagents kit, Applied Biosystems/Thermo Scientific, Foster City, California, US). The PCR reaction volume was 25 *µ*L, containing, 12.5 *µ*L 2X RT-PCR-buffer, 1 *µ*L 25X RT-PCR Enzyme mix, 4.5 *µ*L nuclease-free water, 5 *µ*L of template or nuclease-free water for the negative control and 0.4 *µ*M of each of the primers OPES13A and OPES14A. The primers amplify a 296 base pairs (bp) PCR – product of the 5′ non-coding region (NCR) (Elvander et al. 1998). The thermocycler (ProFlex PCR System, Applied Biosystems, Foster City) was set to the following profile: reverse transcription at 45 °C for 10 min, followed by an inactivation or initial denaturation step at 95 °C for 10 min, and 40 cycles of 95 °C for 15 seconds, 50 °C for 1 min and 72 °C for 1 min, with a final extension step at 72 °C for 7 min. Polymerase chain reaction products were analysed by gel electrophoresis.

### Deoxyribonucleic acid extraction from gels and deoxyribonucleic acid sequencing

Amplified PCR products were extracted from excised gel bands and purified in accordance with manufacturer’s instructions (Thermo Scientific GeneJET Gel Extraction Kit, Thermo Scientific). Samples were subsequently sent to Macrogen Lab in the Netherlands for Sanger sequencing. Sequence data were then entered into the NCBI database for analysis.

### Statistical analyses

All statistical analyses were performed in RStudio version 1.1.383.

### Ethical considerations

Oral consent was obtained from the goat farmers who participated in the study. Firstly, the farmer was informed about the outline and goals of the study, as well as the potential benefits and hazards involved. Farmers were informed that participation was voluntary and they could choose to withdraw their consent at any time. Data protection was achieved through a coding system only known and understood by the people directly involved in the study.

The goats used were subjected to blood sampling with vacutainer needles. If an animal proved difficult to obtain blood from, another goat was chosen instead. No animal was subjected to more than two sampling attempts.

## Results

### Antibody detection

All sampled goats were negative for antibodies to BVDV, and hence, no further testing was performed. In tested cattle, 53.6% (195/364) were seropositive for border disease virus (BDV) or BVDV on Ab-ELISA on an individual level. Seroprevalence within the tested herds ranged from 16.7% to 97.9% (Table 1). All herds (100%) had at least one seropositive animal. Seroprevalence in the sampled dairy farms was 50.9%, and 55.8 % in beef farms (Table 2). Difference between dairy and beef was not statistically significant (*p* = 0.40, chi-squared test).

**TABLE 1:** The sampled cattle farms and their seroprevalence of bovine viral diarrhoea virus antibodies.

Farm	Seroprevalence	%	95% confidence interval
Gaborone 1 (dairy)	88.1	37/42	74.4–96.0
Gaborone 2 (dairy)	28.8	17/59	17.8–42.1
Gaborone 3 (beef)	97.9	47/48	88.9–99.9
Lobatse 1 (dairy)	58.3	21/36	40.8–74.5
Lobatse 2 (beef)	56.3	27/48	41.2–70.5
Molepolole 1 (dairy)	37.5	9/24	18.8–59.4
Molepolole 2 (dairy)	16.7	1/6	0.42–64.1
Ramatlabama 1 (beef)	25.5	13/51	14.3–39.6
Otse 1 (beef)	46.0	23/50	31.8–60.7

**Total**	**53.6**	**195/364**	**48.3–58.8**

**TABLE 2:** Seroprevalences in dairy and beef cattle and their associated confidence interval.

Production system	Seroprevalence	%	CI (95%)
Dairy	50.9	85/167	43.1–58.7
Beef	55.8	110/197	48.6–62.9

**Total**	**53.6**	**195/364**	**48.3–58.8**

A total of 20 samples (5.5%) got results denoted as ‘doubtful’, according to the cut-off values provided by the manufacturer, and they were considered as negative in the analysis.

### Antigen and virus detection

One sample was positive on Ag-ELISA, which is equivalent to an antigen prevalence of 0.27% (1/364). On subsequent PCR runs, the sample positive on Ag-ELISA only resulted in weak bands, even though the procedure was repeated several times to get a more distinct result. In addition, two other samples that were negative on Ag-ELISA were PCR-positive.

The sample that was positive on Ag-ELISA as well as weakly positive on PCR originated from Gaborone 3 which is the herd that had the highest seroprevalence (97.9%). The two samples that were only PCR-positive originated from Gaborone 1 which had the second highest seroprevalence (88.1%). Seroprevalences in Gaborone 1 and 3 were statistically significantly higher compared to all other herds with logistic regression performed in RStudio using the package lme4, with village and herd controlled for in the analysis (*p*-value < 0.001) (Bates et al. 2015).

### Genetic sequencing

The PCR products from two of the viraemic animals, originating from the same herd, were sent for sequencing. The two products were 338 and 337 base pairs in length. The sequences were identical to each other and most similar to genotype BVDV-1a. The segments showed high sequence resemblance with several BVDV-1 strains in the National Center for Biotechnology Information (NCBI) database. Thetop hit, when sorted after maximum score, was USMARC-53875 (Workman et al. 2015) (Table 3), followed by two NADL strains (Vassilev & Donis 2000). The top hits originating from the African continent were five strains from Mozambique isolated in 1991 and 1992, and three strains isolated in South Africa (Baule et al. 1997). These African strains were also the top hits when sorted after identity.

**TABLE 3:** Information from the National Center for Biotechnology Information Blast consolidated into a table.

Strain	Genotype	Max score	Identity	E-value	Origin
USMARC- 53875	BVDV-1	473	95%	9.00E-130	US
Ncp NADL	BVDV-1	473	96%	9.00E-130	US
NADL	BVDV-1	473	96%	9.00E-130	US
M278A/91	BVDV-1	420	98%	1.00E-113	Mozambique
M589A/92	BVDV-1	414	98%	6.00E-112	Mozambique
M139B/91	BVDV-1	414	98%	6.00E-112	Mozambique
S-ALT7/K	BVDV-1	412	97%	2.00E-111	South Africa
USMARC-60765	BVDV-2	231	87%	7.00E-62	US

Note: This table is sorted in accordance to the maximum score appointed by the National Center for Biotechnology Information database. The three strains with the highest maximum scores are included in the table, followed by African isolates with the highest maximum scores. African isolates shared the highest identity similarities with the strains sequenced in this study. A BVDV-2 strain is included for reference.

BVDV, bovine viral diarrhoea virus; US, United States.

## Discussion

Because of the potentially severe consequences of BVDV introduction into a herd, achieving control of the virus is desirable. To accomplish this, knowledge of pathogen prevalence is crucial. In this study, the antibody prevalence in cattle was 53.5% and the viral prevalence 0.83%, as assessed by Ab-ELISA and PCR, respectively. According to Houe and Meyling (1991), antibody and antigen prevalence in endemic regions is usually 60% or more and 1% – 2% or more, respectively. However, these numbers vary greatly with differences in management strategies, for example, trade routines and usage of communal pastures. Previous studies conducted in southern Africa have found antibody prevalences ranging from approximately 10% – 50% in asymptomatic cattle (Depner et al. 1991b; Handel et al. 2011; Hyera et al. 1991; Nigussie et al. 2010; Njiro et al. 2011). These comparatively low prevalences are perhaps more likely because of differences in management strategies, for example, less intensive production systems, rather than the virus not being endemic in the region. It is also important to take into consideration that the study design was not randomised. The selection of both villages and farmers was convenience-based, and therefore, caution should be exercised when extrapolating the results to the target population. However, as the result in this study is comparable to prevalence studies conducted in other countries in southern Africa, the authors believe them to be representable.

The cattle samples analysed in this study were obtained as part of another research project (Ramabu et al. 2018). Because of this, it was not recorded whether the farmers vaccinated their animals for BVDV. It can therefore not be excluded that the detected antibody prevalence is because of vaccination rather than natural infection. However, in Gaborone 1 and 3, antigen-positive animals were found, which indicates active infection. The seroprevalences in these herds were 97.9% and 88.1%, respectively. Rigorous vaccination strategies can give rise to such high percentages, but as viral antigen and nucleic acids also were detected, contact with viraemic individuals is a more plausible cause.

The Ab-ELISA that was used in this study detects antibodies for both BVDV and the closely related BDV, which is also capable of infecting both cattle and goats (Nettleton 1990). Because of this, it cannot be said for certain whether seropositive animals had antibodies to BVDV or BDV. However, viruses from two of the viraemic animals were sequenced as BVDV-1a. The third was positive on Ag-ELISA in addition to PCR, which is specific for BVDV. Therefore, the observed seroprevalences in these two herds are probably because of either the presence of persistently infected animals or active infection with BVDV. Also, to the authors’ knowledge, BDV has never been detected in southern Africa. However, this may be because of a lack of surveys and/or failure of detection, rather than the virus being absent in the region.

When utilising the Ab-ELISA, the negative control was continuously invalid. Reportedly, this was a common problem with the ELISA reader that was used for the analysis. However, for the Ag-ELISA, the same ELISA reader was utilised without similar problems. Both procedures were performed by the same person and using the same basic techniques. The problem with the negative control in the Ab-ELISA may have resulted in antibody prevalence values lower than the actual value. To calculate the percentage of inhibition, the sample absorbance value was divided with the mean of the two negative controls, and then multiplied with 100. Therefore, with low negative control values, the percentage of inhibition value may get misleadingly high and therefore falsely classify samples as negative or doubtful when they were, in fact, positive.

Ideally, when an Ag-positive animal is detected, testing should be repeated after 3–4 weeks to differentiate between acute (transient) and persistent infections. Because of the time-lapse between sampling and analysis, it was not possible in this study. Here, Ag-ELISA was only performed on seronegative animals in herds with at least one seropositive animal to identify persistently infected animals, characterised by being viraemic without seroconversion. However, an animal can be immunotolerant, and hence seronegative to one strain, but seropositive to a heterologous strain because of vaccination or natural infection. Because of this, it is possible that some viraemic animals were missed. However, according to Lindberg and Alenius (1999), this risk is likely negligible.

Six of the antibody negative samples were not subjected to Ag-ELISA and PCR because the sample volumes were too low. Five of these samples originated from Lobatse 2, where seroprevalence was 60%, and one from Ramatlabama 1, where prevalence was 25.5%. According to Houe et al. (1993), seroprevalence in herds with persistent infection is usually 90% or more. It is therefore unlikely that any of these individuals were persistently infected. Also, there was not enough available material to perform RNA isolation and PCR on all of the seronegative samples. Therefore, RNA isolation and PCR were only performed on antibody negative animals in herds with an antibody prevalence of 50% or more. However, within-herd seroprevalence is influenced by a number of factors, for example, management strategies, such as contact between different groups of animals. As this information was not available for the tested cattle herds, a lower cut-off value was chosen to minimise the risk of a viraemic animal escaping identification.

In this study, amplicon obtained from two PCRs was sequenced, and found to belong to the BVDV-1 genotype, which according to previous studies is more common in Africa than BVDV-2 (Baule et al. 1997; Emran et al. 2014; Kabongo, Baule & Van Vuuren 2003; Thabti et al. 2005; Ularamu et al. 2013; Van Vuuren 2005; Vilcek et al. 2000; Yesilbag et al. 2017). For example, in 2013, 82.5% of samples obtained from feedlots in various parts of South Africa and Namibia belonged to BVDV-1, and 17.5% to BVDV-2 (Ularamu et al. 2013). The two isolates sequenced in this study were identical to each other, which is not surprising, given the fact that they originated from animals in the same herd. The two segments were similar to a high number of sequences in the NCBI nucleotide database besides the ones discussed here. This is not surprising either because the sequenced part of the genome, that is, the 5′NCR, is a highly conserved region (Bauermann et al. 2013; Van Vuuren 2005).

The sequence that shared the most similarities, according to the NCBI nucleotide database, when sorted after maximum score, is USMARC-53875, followed by two NADL strains (Vassilev & Donis 2000) (Table 3). NADL is a reference strain for BVDV-1a that originates from North America (Vassilev & Donis 2000). The most similar sequences from the African continent originate from Mozambique and South Africa. According to a review by Yesilbag et al. (2017), BVDV-1a is the BVDV variant most often detected on the African continent.

In the goats sampled in this study, no evidence of antibodies to BVDV or BDV could be found. Previous studies conducted in other African countries have found seroprevalences of 5% in neighbouring Namibia (Depner et al. 1991b). In Tanzania, different studies have recorded prevalences ranging from 1.7% to 24.9% (Hyera et al. 1991; Torsson et al. 2017). All goats sampled in this study originated from herds around Gaborone, which is the same region as the viraemic cattle were found. Because of this, it is unlikely that the 0% seroprevalence is because of BVDV not existing in the area. It is more likely that the goats were insufficiently exposed to the virus to develop antibodies. Also, seroprevalence in goats is in general considerably lower compared to cattle. This is probably because persistent infection is an unusual occurrence in this species (Bachofen et al. 2013; Broaddus et al. 2007; Depner et al. 1991a; Passler et al. 2014).

There is a great need of further research on BVDV in Botswana. More knowledge is needed on the implications of BVDV in these settings and its economic significance to smallholder farmers. Such studies should not only focus on the direct effects of BVDV but also on its role as an immunosuppressing co-pathogen in, for example, respiratory and enteric diseases. Also, more extensive knowledge is needed on prevalence, as well as local risk factors for transmission, because this knowledge is imperative when establishing efficient and cost-effective measures for viral control. Last but not least, there is a great need for extended knowledge regarding control methods applicable to the local settings. This coupled together could lead to a reduced incidence of BVDV, and thereby not only improve animal health and welfare, but also increase profits for farmers in Botswana.

## Conclusion

Seroprevalence of BVDV in and around Gaborone was 0.0% in goats and 53.6% in cattle. The seroprevalence was 50.9% in dairy cattle and 55.8% in beef cattle; however, this difference was not statistically significant. The two herds from where the three viraemic animals originated had significantly higher seroprevalence compared to the other herds. Also, two of the detected viruses belonged to the genotype BVDV-1a.
